# Isotope Effects in ESR Spectroscopy

**DOI:** 10.3390/molecules18066679

**Published:** 2013-06-07

**Authors:** Reinhard Stößer, Werner Herrmann

**Affiliations:** 1 Institute of Chemistry, Humboldt University of Berlin, Brook-Taylor-Str. 2, Berlin 12489, Germany; E-Mail: rs1939@gmx.net; 2 Institute of Pharmacy, Free University Berlin, Kelchstr. 31, Berlin 12169, Germany

**Keywords:** effects of nuclear spin, mass dependency, magnetic moment, local symmetry, time dependency, vibronics and tunnelling in ESR

## Abstract

In order to present the relationship between ESR spectroscopy and isotope effects three levels are considered: (i) ESR spectroscopy is described on a general level up to the models for interpretation of the experimental spectra, which go beyond the usually used time and mass independent spin-Hamilton operator, (ii) the main characteristics of the generalized isotope effects are worked out, and finally (iii) the basic, mainly quantum mechanical effects are used to describe the coupling of electron spins with the degrees of freedom, which are accessible under the selected conditions, of the respective paramagnetic object under investigation. The ESR parameters and the respective models are formalized so far, that they include the time and mass depending influences and reflect the specific isotope effects. Relations will be established between the effects in ESR spectra to spin relaxation, to spin exchange, to the magnetic isotope effect, to the Jahn-Teller effects, as well as to the influence of zero-point vibrations. Examples will be presented which demonstrate the influence of isotopes as well as the kind of accessible information. It will be differentiated with respect to isotope effects in paramagnetic centres itself and in the respective matrices up to the technique of ESR imaging. It is shown that the use of isotope effects is indispensable in ESR spectroscopy.

## 1. Introduction

The physical explanation of the phenomenon and the usage of isotopes for industrial or medical purposes are highly developed technical and scientific areas [1,2]. This is also true for the method of electron spin resonance (ESR) [3,4]. ESR supplies a well-developed instrumental as well as a theoretical basis. The ESR method is suitable to examine systems with unpaired spins [3,5], which interact with each other, as well as with stationary or time-dependent magnetic and electric fields, coherent electromagnetic radiation (in general microwaves), and the magnetic moments of neighbouring nuclei. On this the microscopic degrees of freedom of the system under examination act mediating and modulating in addition to the local structure constraints. Based on the experimental findings and the respective theoretical interpretation many basic phenomena of quantum mechanics can be understood. Moreover, important contributions to the solution of basic problems of physics, chemistry, biology, medicine, geology, and environmental science could be delivered. The method is indispensable on examining the effect of ionizing radiation of foodstuff and other objects. This requires the cooperation of scientists from different fields of work. The difficulty of the method is and will remain the complex interpretation of the experimental findings, even on considering the technical progress in the field. Essential problems of ESR can be solved by the use of isotopes. Last but not least the application of the isotope methods in ESR requires good skills in the art of chemical preparation and a certain “chemical feeling” for discussing the results.

Moreover, using isotopes in spectroscopy has been done for a long time already, and is highly developed nowadays [1]. A large number of publications speak for that fact. Finally, it is the task of spectroscopy to deliver information about the system under investigation. Applications in mass spectrometry, infrared spectroscopy, and nuclear magnetic resonance (NMR) prove that statement. Altogether, the thermal, mechanical, electrical, optical, magnetical, and chemical properties of the samples, including their enclosed atoms, are specifically changed on using isotopes.

The usage of isotopes in ESR spectroscopy supports the signal assignment and, thus, to obtain the desired physical, chemical, biological, and geological information about the examined systems out of the recorded spectra. Basically there are three fields of application on using isotopes: (i) Proof and application of quantum effects by means of ESR, (ii) isotopes as means for signal assignment in case of complex ESR spectra, and (iii) detection and interpretation of kinetic and isotope effects under participation of the electron spin. Concerning the topic “isotopes as a means for the identification in the case of complex ESR spectra” one can find a large number of papers, *i.e.*, the respective methodology has been developed farthest, while publications in the other two fields are relatively rare due to their higher theoretical standard.

The usage of isotopes may significantly contribute to simplify complex spectra, to explain the origin of specific atoms in chemical products, or to allow the observation of structural and micro-dynamic processes, respectively. This is based on the characteristic differences in the properties of isotopes such as mass, nuclear volume, nuclear spin, and nuclear magnetic moment. Using these differences in properties requires good skills in chemical synthesis for the defined placement of isotopes in the molecular structure or in the lattice of a solid in case there is no possibility to use the natural abundance.

All this is connected to the underlying theoretical models such as Jahn-Teller effects, tunnel effects, vibronic couplings, zero-point oscillations, spin exchange, spin-phonon couplings, and magnetic isotope effects.

The detection and identification of paramagnetic species in ESR is still a problem, e.g., in order to prevent fast chemical reactions many radical systems have been made observable at low temperatures or in a chemical way by introducing steric hindrances into their structures (spin probes, spin traps [6,7]). Simultaneously there has been a fast technical progress in the ESR equipment particularly with respect to sensitivity and spectral resolution [3,8,9,10,11,12,13]. Nevertheless the isotopic marking is of great importance for the decryption of the hyperfine structure patterns.

**Figure 1:**
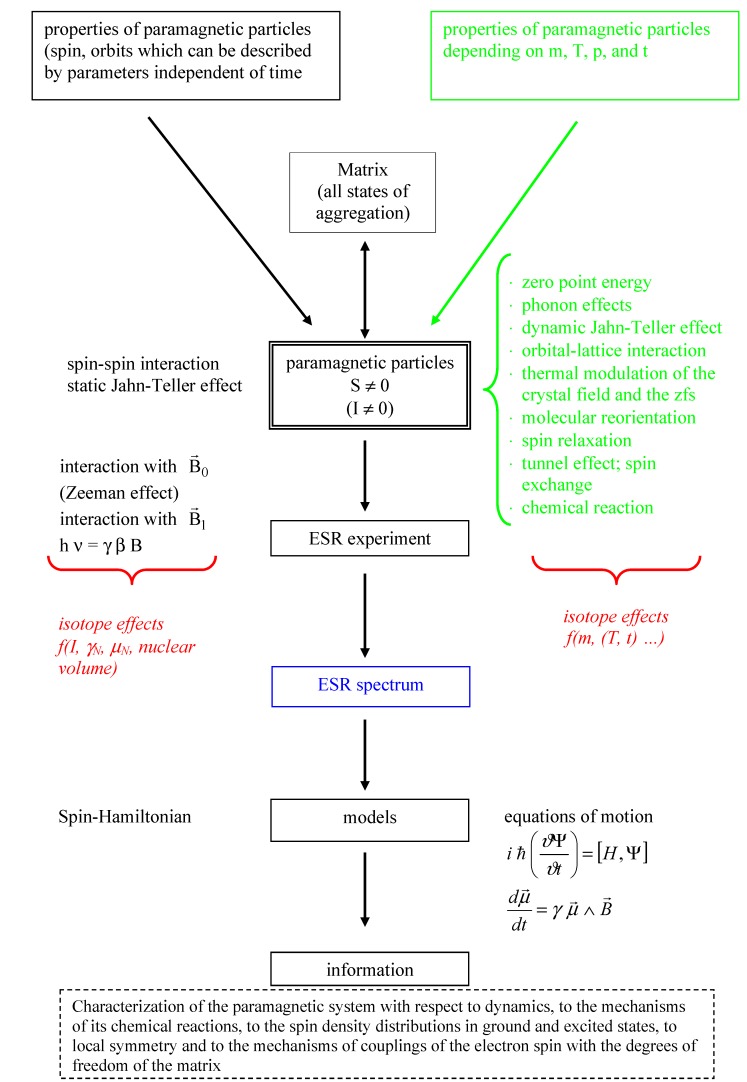
Schematic representation of the realization of an ESR spectrum (blue) starting from the different properties of a paramagnetic particle including different internal and external interactions as well as specific influences of the matrix. The green marking shows those parameters including the resulting effects which are not part of the common Spin-Hamiltonian. The possible isotope effects are marked red. Using the given models for interpretation detailed information about structure and dynamics of the examined paramagnetic systems will be accessible.

Thus, it is an aim of the present paper to present the ‘state of the art’ of ESR (somehow deviating from the existing monographs [3,14,15,16]) in such a way that the relations between the ESR parameters and the characteristic properties of isotopes become evident. These relations will be discussed by means of selected properties and suitable examples starting with the schematic representation of the interactions of a paramagnetic particle (see Figure 1).

## 2. Electron Spin Resonance (ESR) and Relations to Isotope Effects

### 2.1. Basic Principle of ESR

ESR is the most important analytical instrument for the examination of paramagnetic compounds. At first this is related to the structure of molecules, distributions of spin densities [17,18] and the dynamics of molecules. There are many applications of the aspects, which will be discussed in greater detail further on, in the fields of biology, medicine, the chemistry of radicals [6,7], metal-organic compounds, solid state physics and chemistry, food chemistry, pharmacy, in geology, and archaeology.

ESR differs from nuclear magnetic resonance (NMR) only quantitatively [19]. It belongs to those spectroscopic methods which apply magnetic fields and coherent electromagnetic radiation. Usually the high-frequency susceptibility χ”(ω) of a sample is measured in a device in which the high-frequency magnetic induction 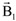 is perpendicular to the static field 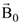. According to the resonance condition:
h ν = g β 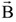 or ω = γ 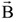
(1)
where γ: gyromagnetic ratio, the internal magnetic moments (of the unpaired electrons) will flip. Thus, the ESR experiment consists of microwave radiation entering a cavity (with a sample) which is critically coupled to the microwave generator and absorbs the microwave completely. On passing the resonance, e.g., by variation of the static field 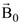, a mismatching of the resonator and hence, and a part of the microwave radiation will be registered at the microwave diode.

**Figure 2:**
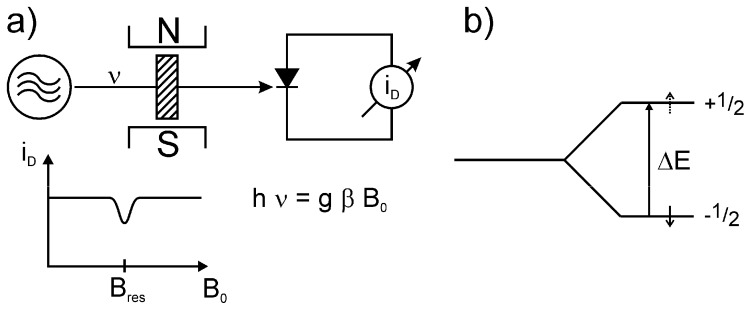
Strongly simplified scheme of an ESR spectrometer (**a**) and splitting of the nuclear spin states with I = ½ in the zero field by the electron spin S = ½ (↓) (**b**).

Figure 2(a) shows a simplified scheme of an ESR apparatus. The sample is located between the poles on an electromagnet and will be exposed to microwave irradiation of frequency ν. Plotting the microwave diode current i_D_ versus the magnetic induction B_0_ gives a horizontal line with a dip in the case of resonance (h ν = g β B_0_), which is an expression of the microwave absorption by the electron spins of the sample at B_res_.

From a theoretical point view this means that if the energy levels and the wave function of a paramagnetic system are known, the disturbance of the system by a magnetic field (Zeeman effect, [3,20,21]) and transition between the Zeeman levels are the precondition for the ESR. Then that and the resonance absorption of electromagnetic radiation in the microwave or radio frequency (RF) range together with spin relaxation and other temperature and pressure depending effects (see Figure 1) determine the resulting ESR spectra [3,19]. Generally many ESR spectra can be described by a simple Spin-Hamilton operator (see [3,22]). Its matrix elements determine position and intensity of the absorption lines in the microwave or RF range of the electromagnetic radiation. But explicit mass and time dependent effects are ignored and only a part of isotope effects (magnetic moments 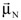
and nuclear spins 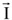) can be taken into account. To grasp the full spectrum of isotope effects further models (see Figure 1 and Section 2.9.) are necessary. The electron and nuclear spin (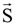 and 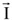) as well as the magnetic induction 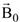 (and their combinations; see Figure 1) are represented by operators connected by spin coupling coefficients [17,18], which depend on structure and properties of the examined system (see e.g., [23,24]). Favourably the arrangement of the Spin-Hamilton terms will be selected in such a way that they transform as the irreducible presentation of the point groups of the system [25]. Next to the experimental possibility to determine the components of the Spin-Hamiltonian (see e.g., [3,17,18,22]), they can be calculated by quantum mechanical means on using electronic state (or wave) functions. As mentioned above the Spin-Hamiltonian described in such a way does not depend on m, T, p, n (n: Amount of substance), …, phonons, *etc.* It is assumed, that on applying that spin-Hamilton model the atomic nuclei are “fixed”. On doing so, vibronic effects cannot be considered explicitly, despite the fact that they may change experimental spectra considerably. This is true, e.g., for the Jahn-Teller effect [25], which in ESR spectroscopy is manifested in vibronic reduction, tunnel splitting, random-strain, and relaxation effects, and causes e.g., a distinctive dependency of fine and hyperfine structure on the temperature. These phenomena get eye-catching e.g., on observing the un-common paramagnetic resonance properties of Cu(II) compounds [25,26]. A qualitatively correct interpretation of the respective spectra can be achieved as a function of the Jahn-Teller dynamics of the nuclear configuration (and therefore the nuclear masses, too [25]).

Therefore there is a necessity for a considerable extension of the original Spin-Hamilton operator for the interpretation of real ESR spectra, including the consideration of isotope effects. Before that a deeper understanding about the phenomena and properties of electrons and nuclear spins should be developed. Under aspects of applications, electron and nuclear spins have to be considered as follows:

(i) Taking into account the Pauli principle in form of quantum numbers inclusive the spin conservation, e.g., in chemical reactions, during Pauli repulsion (see Section 3.8.11.), and finally in anti-symmetry of the electronic wave function. Due to the vector character of the spin the couplings with the vector components of electric and magnetic fields are to represent by coupling matrices with tensor character. If these matrices are determined (ĝ,Â,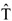, …, see [3,27]), important information about structure and dynamics of the paramagnetic species can be gained on using further models (see Section 2.8. and Section 2.9. and Figure 1). Simplified the spin can be considered to be a further indicator of the total state (wave) function [17,18]. Contrary to the indistinguishable electrons having only one spin quantum number S = ± ½ (see also [17,28]), the nuclei including the isotopes are described by a considerable number of nuclear spins and the corresponding magnetic dipole and electric quadrupole moments. Together with this a large number of applications exist which are based on nuclear moments. To name only the most important one: nuclear magnetic resonance (NMR). Which effects or processes explicitly involve spins or which observables are produced by the spin? There are two more items to be named:

(ii) The electron-spin interaction leads to a splitting of the originally degenerated energy levels and the transitions between them represent the fine structure (see also Section 2.3.).

(iii) It is not always necessary to solve the complete many-electrons equation of motion (e.g., the complete Schrödinger equation) in order to achieve an acceptable adaption to the experimental fine structure. In most of theses cases the effects of spins are essential and a Spin-Hamilton operator can be established which is (only) a function of the spin operators [17,18]. Thus, the Spin-Hamilton operator connects theory and many of the spin depending experimental results.

By means of ESR one could contribute to answering basic questions in physics, chemistry, geology, archaeology, biology, and medicine [3,29]. That means in particular, the detection and characterization of paramagnetic states including their surrounding in all states of aggregation, mainly in fluid systems, crystalline and amorphous insulators and semiconductors. The majority of the up to now presented papers is related ground state phenomena, but important and in part unique information about optically excited states (e.g., triplet states [3,27]) can be obtained, too.

The application of spin probes allows the characterization of chemically and biologically important systems concerning their structures, dynamics, and function [6,7]. It has to be emphasized, that investigations of the mechanisms of photosynthesis, other biochemical, as well as chemical reactions have been carried out on using the techniques of spin trapping and spin polarization [6,7,30]. Unique contributions to the consequences of the Jahn-Teller [25,31] and tunnel effects [32,33] could be provided by ESR spectroscopy. The phenomenon of spin exchange [34,35] is an important field of ESR because it not only allows the characterization of the matrix but also the differentiation between chemically reactive and non-reactive collision of particles.

Last but not least, ESR delivered proofs for basic theories in physics and chemistry, as well as for the existence of quantum effects (see e.g., Pauli principle, Hund’s rule, e.g., the quartet ground state of N, exchange interactions [36]). For that the usage of isotopes plays an important role which should not be underestimated (see Sections 3.3.2. and Sections 3.8.1.1.).

The technical progress contributed much to raise the information content of ESR spectra mainly through higher sensitivity, the usage of non-stationary methods (e.g., pulse methods [14,15]), multiple frequencies, multi-dimensional procedures, imaging, and finally the complete field of chemical means for spectra interpretation (use of isotopes, introducing steric hindrances, trapping techniques).

Nonetheless, the usage of isotopes remains of great importance for improving the spectral assignments despite the latest technical progress in electronic equipment and the development of (new and improved mathematical) models, and there is no sign for a replacement of isotope usage. This will be clear on looking e.g., at the important element oxygen. The direct ESR detection of the paramagnetic molecule is still difficult [3]. In compounds it can be observed only in case of its isotope ^17^O (I = 5/2, 0.0373% natural abundance) directly. Other spectroscopic methods (NMR, MS, IR, *etc.*) have to use isotopic patterns for a doubtless interpretation of their results, too.

It should be mentioned here, that the usage of isotopes as replacement of the naturally occurring atoms is expensive. Moreover, the synthesis of isotopically labelled compounds requires high technical skills.

### 2.2. Parameters of the Spin-Hamiltonian

#### 2.2.1. The ĝ Tensor, the Interaction of Spin and Orbital Moments with the External Magnetic Induction 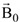


The electronic ĝ tensor represents the coupling of the components magnetic induction 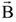 and electron spin 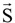 [see Equation (2)]. With respect to its transformation behaviour that coupling element (coupling matrix) is a tensor object. It mainly determines the Zeeman term:
H_Zeeman_=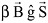
(2)

Under a physical aspect, ĝ is determined by the orbital momentum 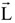 (see quenching of orbital moments in molecular systems [3,17,18,23,24]) as well as the kind and energy levels of the excited electronic terms.

The frequently determined “mean value” of ĝ mainly inform about the nature of the participating heavy atoms in the paramagnetic centre. For free electrons a value of g_e_ = 2.0023 was determined, while nitroxyl spin probes have value around 2.05 [6,7,37], and for heavy ion like Cu^2+^ it will be around 2.2 [38].

In comparison to NMR the large difference in mass between the electron and the nucleus is obvious and results in chemical shifts of some ppm in NMR, whereas it might be in the percentage range in ESR. The difference between the variation range of the g-factor and the NMR chemical shift is based on different facts: The magnetic moment is indirect proportional to the mass whereas the chemical shift is the result of the interactions with the electron shell. This interaction is dominated by electron states with non-zero orbital moments in the case of the g-factor and is much smaller for the shielding effect of the nuclei because the effects of higher electronic states reach the nuclei only by spin polarization effects. But it should be mentioned that the isotopic influence on the ĝ tensor is relatively small (see Section 3.2.). The nuclear ĝ factor is determined by the respective nuclear properties and is specific for any nuclear paramagnetic isotope [1,39].

#### 2.2.2. The Hyperfine Interaction

##### 2.2.2.1. Nuclear Spin, Nuclear Magnetic Moment, Hyperfine Structure, and General Relations to Isotope Effects

Due to the fact that the nucleus can be considered to be an electromechanic system it should have electromagnetic moments. The first hypotheses concerning a nuclear spin and the nuclear moments have been made by Allen in 1915, Oxlay in 1921, Pauli in 1924, and others, while Pauli considered the nuclear spin to be reason for the hyperfine structure of optical spectra already [40].

First experimental results came from Back and Goudsmit [41] based in particular on their observations of the bismuth nucleus (see also [28]). A first value for the nuclear magnetic moment (^7^Li) was delivered by Breit and Doerman [42] and Jackson [43] tried to measure the nuclear moment of ^133^Cs directly by means of optical spectroscopy (it is interesting that starting from 1963 the transition between two hyperfine levels of that isotope serves as the unit of time; see also [44]).

In general, by improving the resolution of the atomic spectra it could be shown, that the fine structure again splits (e.g*.*, the further splitting of the doublet of the yellow sodium line by the nuclear spin of sodium) and the originally four quantum numbers of the electronic states (n, l, m, s [45,46,47]) are not sufficient to explain that phenomenon. A nuclear spin had to be assigned to the respective nuclei, too. It turned out that in case of isotope effects the charge distribution in the nucleus is no longer spherically symmetric; the nucleus-energy level is different for different isotopes. Moreover, another isotope effect comes along; the mass dependence of the Rydberg constant [46,47]. That dependency results from a small ‘joint movement of the nucleus’ due to the movement of the surrounding electrons. That effect is largest with the lightweight elements and it was used by Urey [48] for discovering ^2^H (D).

Comparably accurate (and unambiguous) values for the nuclear moments were given by Rabi [49] by means of atomic beam method.

Today the magnetic nuclear moments of all known isotopes have been determined with adequate accuracy [1,50]. In general the nuclear moment is a function of the spin moment and the orbital moment of the nucleus, whereas the nuclear spin and the integral orbital angular momentum contribute to the total angular momentum of the nucleus. The magnetic dipoles of the nuclei contribute to the following phenomena [50]:

(i) hyperfine structure and magnetic splitting of the hyperfine structure in atomic and ESR spectra [3];

(ii) band spectra of homonuclear molecules [1];

(iii) magnetic deflection and resonance with atomic and molecular beams [51];

(iv) resonance and relaxation effects of the nuclear paramagnetism [22];

(v) hyperfine structure of the microwave spectroscopy [52].

The hyperfine structure is caused by two phenomena:

(i) interaction of the electrons with nuclear moments (magnetic dipole and electric quadrupole moments),

(ii) isotopic shifts in the atomic spectra, *i.e.*, a splitting in isotopic mixture occur; there are no effects with individual atoms.

The hyperfine structure is usually around 10^3^ smaller than the fine structure (see Sections 2.3.and Sections 3.4.). Different values of the energy of magnetic interaction correspond to the values of the different mutual orientations of electron and nuclear spin.

The direct determination of the nuclear moments of atoms with unpaired electrons is challenging due to the strong magnetic field at the nucleus caused by these electrons. On the other hand this is advantageous, e.g., to obtain sensitively detailed information about the paramagnetism of the respective sample by means of the magnetic resonance in the GHz range [50,53,54,55,56,57]. Nevertheless it has to be considered that exchange interactions [36] average out the hyperfine structure on magnetically undiluted samples [51].

If the nucleus of an atom in a paramagnetic system has a magnetic moment a magnetic field of some mT will result that acts upon the electrons. The magnitude of the field depends on the orientation of the nucleus, which is usually fixed during electronic transitions. Therefore any transition (or the respective spectral line) will split in 2 I + 1 components. It has to be mentioned that the field strength of ESR at about 10 GHz is about 0.3 T and does not have a strong influence on the orientation of the nuclear moments. Thus the nuclear magnetic moment or the nuclear spin of the respective nucleus can be identified. Gorter [58] developed the hypothesis that beside 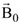 the nuclear magnetic moment plays a role on splitting the ground state of paramagnetic systems. At the nucleus that field should have a magnitude of some 10,000 mT and should cause a hyperfine splitting larger than 0.01 cm^−1^. Penrose [59] delivered the first successful experimental proof of the ESR hyperfine structure. The coupling constant of the hyperfine structure of paramagnetic systems is one of the most important parameters in ESR and from the very beginning it is connected to the different action of isotopes (e.g., ^63^Cu and ^65^Cu) [59,60,61,62]. The hyperfine structure of paramagnetic systems allows the determination of the nuclear spin simply and directly, as well as the relation of the magnetic moments of two isotopes of the same atom.

After Penrose’s discovery the nuclear magnetic moments could be determined by means of ESR as there are ^53^Cr [63], ^143^Nd and ^145^Nd [64], ^159^Tb [65], ^165^Ho [66], ^56^Co and ^57^Co [67], ^235^U [68], ^63^Cu and ^65^Cu [62], and ^60^Co [69] (see also [70,71]).

Another milestone in the sequence of discoveries derived from the hyperfine structure had been the proof of hyperfine interactions in the system Na_2_[Ir(IV)Pt]Cl_6_ H_2_O [72,73]. The careful analysis of the ESR spectra not only revealed the hyperfine contribution which results from the interaction of the unpaired d-electrons and the nuclear moments of the iridium isotopes ^191^Ir (I = 3/2) and ^193^Ir (I = 3/2), but the interaction with the ligand atoms ^35^Cl (I = 3/2) and ^37^Cl (I = 3/2), too. That had been the proof that unpaired electrons are not only located at the central atom. In the following the models of chemical bonding had been refined by assigning covalent contributions [55] (see also [74]).

In general the analysis of hyperfine structures, in particular those of organic radicals and radical ions [75], significantly contributed to the understanding of the phenomena of delocalization of electron and spin densities in molecules and their (quantum mechanical) interpretation [3,18,19]. Spin density as the difference of electron densities with α and β spin [18] can also have negative values contrary to electron densities.

Further on, by means of the hyperfine findings the spin and exchange polarizing action of unpaired valence electrons or outer shell d and f states (*i.e*., electrons whose magnetization disappears formally near the nucleus) onto the 1s states and, thus, the hyperfine fields at the nucleus could be discussed [18,19,76]. Actual ideas concerning the chemical bond are given in [74].

Real atomic nuclei have a finite mass, a charge, which is distributed over a finite volume, and (possibly) a nuclear spin I different from zero. Due to that magnetic dipole moments and electric quadrupole moments result, which couple to an overall moment. That property influences the atomic energy level slightly (about 10^−5^ Rydberg [45,46,47]) but measurably. The finite mass leads to a “mass shift” and the finite volume for the charge to a “volume shift” (some ppm) of the spectral lines, which at best can be observed due to their isotopic shifts. With that elements with atomic numbers <40 deliver a positive shift and elements >60 negative shifts. The mass shifts give positive and the shifts in nuclear volume negative contributions [32,33,76]. On the other hand the magnetic dipole and the electric quadrupole moments lead to hyperfine structure, which is based on the respective splitting of the energy levels. Today the importance of the coupling of magnetic moments and the surrounding unpaired electrons goes beyond the determination of nuclear moments or structural and dynamic details of atomic und molecular states, respectively. Due to the influence of the hyperfine structure onto the optical selection rules and the transfer of moments, e.g., from light to atoms, laser experiments allow the manipulation of atoms by means of light. The hyperfine structure is of greatest importance in the case of free and bound atoms which have unpaired electrons. Particularly via these interactions several isotope effects can be detected or used, respectively. According to the formula for the Bohr energy levels the energy increases in the state n with increasing mass:

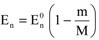
(3)
where m: mass of the electron; M: mass of the nucleus

Particularly for spectra of systems resembling that of hydrogen, the mass dependent isotope effect may be interpreted considering the reduced mass 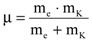 instead of the real mass (9.109 × 10^−31^ kg). In a descriptive way this can be explained by a joint movement of the nucleus (proton mass: 1.672·× 10^−27^ kg, muonium mass: 9.106·× 10^−31^ kg.

The influence of the nuclear volume on the hyperfine interaction will be explained in the following sub-chapter (under “static (stationary) hfs”) and [77].

###### 2.2.2.1.1. Phenomena of Magnetic Hyperfine Structure Exemplified on the Pair of Isotopomers 1H and 2H

As mentioned above the hyperfine structure (hfs) [78] is one of the most important parameters in ESR spectroscopy. Due to the interactions of electron spin 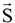
and nuclear spin 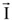 energy levels of the electron split.

This interaction involves electronic as well as nuclear properties. In the simplest case the splitting a depends on the nuclear parameters g_N_ (nuclear g factor), β_N_ (nuclear magneton), and the spin density |Ψ(0)|^2^ at the nucleus:

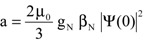
(4)
here μ_0_: magnetic field constant. By means of a well-known pair of isotopomers, hydrogen ^1^H and deuterium D or ^2^H, the differences in hyperfine coupling (hfc) based on the nuclear constants (Table 1) shall be shown.

**Table 1:** Selected physical constants of ^1^H and ^2^H.

	^1^H	^2^H
nuclear magnetic moment	μ_H_ = g_N_ β_N_ I = 1.4106 × 10^−26^ J T^−1^	μ_D_ = g_N_ β_N_ I = 1.4106·× 10^−26^ J T^−1^
nuclear spin	I = ½	I = 1
nuclear g factor	g_H_ = 5.5856	g_D_ = 0.8574
gyromagnetic ratio	γ_H_ = μ_H_/I h = 2.6752 × 10^8^ s^−1^ T^−1^	γ_D_ = μ_D_/I h = 4.1067 × 10^7^ s^−1^ T^−1^

In a good approximation the ratio γ_i_/γ_j_ for two isotopomers i and j can be obtained via the ratio of the experimental hyperfine coupling constants a_i_ and a_j_. E.g., from the experimental ratio a_H_/a_D_ = 50.685/7.780 = 6.514 follows γ_H_/γ_D_ = 6.514.

Generally that ratio may be larger/smaller than 1 depending on the degrees of freedom of the respective paramagnetic molecule. In particular the zero-point vibration delivers about details of the structure, e.g., the distribution of the spin density.

###### 2.2.2.1.2. Static and Dynamic Aspects of the Hyperfine Interaction

(i) Static (stationary) hfs:

Within the respective ESR spectrum the following hyperfine phenomena can be observed: isotropic and anisotropic (Fermi contact and dipolar) contributions [17,18], number of lines and ratio of intensities (as function of the number of involved atomic nuclei and their nuclear spins), and values (and sign, respectively) of the hyperfine coupling constants. The stationary hfs is an important mean to identify the kind of paramagnetic species. This is mainly related to stable (or stabilized) radicals in solutions and the solid state, paramagnetic defects in the solid state, in transition metal ions in chemical and biochemical processes, as well as in optically excited electronic states. Then the influence of the isotopes is here mainly related to differences in the nuclear moment μ_N_, the nuclear spin I, and the nuclear volume. The importance of the nuclear volume for the hyperfine interaction results from the following considerations: The electrostatic potential inside a nucleus is reduced and does not behave like Ze/r. If the valence electrons penetrate into this region, especially the s electrons, then their energy will become larger because of the reduced potential. In general the charge distribution is spread out if neutrons are added to a nucleus because the energy is raised further. Therefore a reduction of the binding energy results and the transition energy is decreased. It means a negative mass shift can be observed if the s electrons represent the lower energy state at the transition [77]. The resulting volume correction is given by Sobel’man *et al*. [79]. Moreover, for reducing the line width, caused by magnetic dipole interactions with atoms of the matrix, the isotopic substitution may be favourable, too (e.g., exchange of hydrogen by deuterium in K_2_Cu(SO_4_)_2_·H_2_O).

(ii) Dynamic hfs effects:

These effects can be recognized easily by means of the (mostly temperature and concentration depending) changes of the line width of the cw spectra. More detailed information can be obtained by non-stationary ESR techniques (see e.g., [14]). These effects are caused by e.g., spin-rotation interactions, molecular reorientation diffusion [6,7], dynamic polarization of 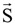
and 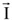 [30,34,35], spin exchange [35], dynamic Jahn-Teller effect [25], tunnel effect [33,80], vibronic coupling, and others. Under a dynamic aspect the isotope effects act upon the zero-point energies by vibrational averaging with amplitudes depending on isotopes. Morton and Preston used this effect for investigating the hyperfine structure of F_2_NO [81].

### 2.3. The Zero-Field Splitting

By means of ESR the phenomenon zero-field splitting (zfs) [3,27] can be observed with systems with S≥1; *i.e.*, there will be a splitting of the originally degenerated spin states (in spherical symmetry) already in the absence of an outer magnetic field (B_0_ = 0) due to internal magnetic (dipole and spin orbit) interactions. These splittings, giving the ESR fine structure, react sensitively on very small changes of the symmetry of the electric field (e.g., of a coordination polyhedron) in which the unpaired spins are located (see also [82]):

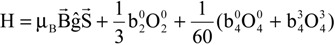
(5)

The corresponding spin-Hamilton operator 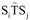, (collecting magnetic dipolar and exchange spin-spin interactions) which is usually used for the parametrization of the ESR fine structure [Equation (5)], does not contain a mass depending term. Thus, for the description of the respective influence of isotopes the interaction with phonons have to be considered, e.g., in form of the spin-phonon operators [29,83] as it was shown by Shrivastava [84] for the system ^52^Cr, ^53^Cr in the system MgO:Cr^3+^.

### 2.4. Spin Relaxation

Spin relaxation processes generally belong to the family of processes in which a retarded reaction of the system proceeds after the end of an outer action. That means for spin systems, a process in an (originally) magnetically ordered system returns in a state of equilibrium. E.g., experiments on adiabatic magnetic cooling have shown that a sudden process of demagnetization in an ensemble of electrons (S = 1/2) in a crystal lattice leads to an increase of the entropy ΔS. In other words: that ΔS takes up a certain part of the thermal energy of the lattice vibrations produced via interactions with the spins. In this connection, it is of interest to know how much time it takes for that exchange of thermal energy [4] after the thermal contact of the ensemble of “electronic magnets” to the degrees of freedom of the vibrations of the recent surrounding. That process will be named the spin-lattice relaxation, while the phonon system (elementary excitation of lattices oscillations) plays an important role [29,83,84]. Under a mechanistic aspect the interaction proceeds via modulation of the electric field due to the vibration of the neighbours with the consequence of the modulation of “orbital movement”. These processes depend on the mass of the involved atoms, and therefore give rise to isotope effects in ESR.

**Figure 3:**
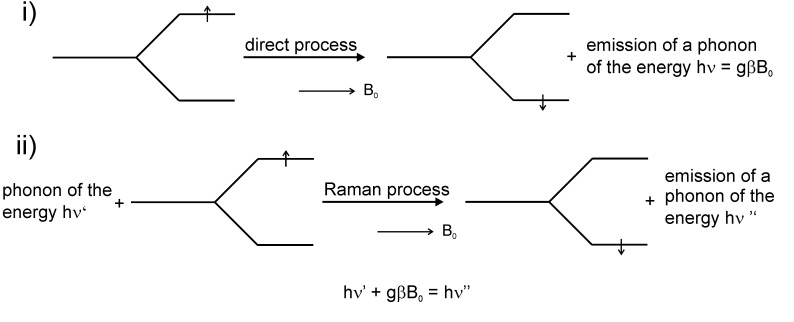
Mechanisms of spin-lattice relaxation.

If one considers the spin-lattice relaxation with a time constant T_1_ (or other models [3,4]) two important mechanisms have to be kept in mind; (i) direct and (ii) indirect (Raman) processes (Figure 3). In process (i) a spin of m_S_ = +1/2 and the energy E = ½ gβB_0_ changes into the state m_S_ = −1/2 and the energy E = −½ gβB_0_ under emission of a phonon of the frequency ν =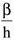
gB_0_. Process (ii) is characterized by the action of a phonon of the energy hν’ on a spin with m_S_ = +1/2 and E = ½ gβB_0_ and in a non-elastic scattering process the spin state changes to m_S_ = −1/2 and energy E = −½ gβB_0_ under emission of a phonon with the energy hν”. The following energy results: hν’ + gβB_0_ = hν” [4].

The paramagnetic relaxation is mainly determined by the spin-phonon interactions in paramagnetic crystals [85,86]. The oscillations of the neighbouring atoms and atomic groups, respectively, lead to the modulation of the orbital moments of the unpaired electrons and, thus, to the induction of spin transitions via spin-orbit couplings. The respective theoretical basic ideas have been developed by Waller in 1932 [21] already.

An example for the influence of isotope marking, *i.e.*, for the shift of the fine structure parameters, has been given by Shrivastava [84] by means of ^52^Cr and ^53^Cr doted MgO. Based on an effective spin-phonon operator he was successful in a correct interpretation of observed isotope effects.

Within the Bloch model the second kind of spin relaxation is named transversal relaxation.

Differently to the longitudinal relaxation, which is characterized by releasing energy to the surrounding matrix (e.g., crystal lattice), spin-spin relaxation means the exchange of energy by spins of similar type. This causes the homogenization of the temperature of a spin system within a characteristic period of time T_2_.

E.g., a nucleus which returns from the excited spin state releases energy to a neighbouring nucleus which gets excited then. With this only the phase relation of the participating spins changes (for applications see Section 3.8.2.1).

### 2.5. Spin Exchange

On colliding of atoms or molecules with incompletely occupied electronic states (unpaired electrons), respectively, the process of spin exchange may occur [35]:

A(↑) + B(↓) Ý A(↓) + B(↑)(6)

In doing so, the hyperfine state of the colliding particles will change [32]. Spin exchange can be considered as an example of tunnelling exchange of electrons (see 2.6.). It is a process where electrons of two atoms (or molecules) are exchanged via simultaneous tunnelling in opposite directions. E.g., if one has a look onto the spin exchange of the collision of two particles with 1s electrons (e.g., hydrogen atoms), the wave function of the particles with opposite spins (until collision) can be written as follows:
ψ (t→−∞) = (α_1_ β_2_ φ_a_ (1) φ_b_ (2) − α_2_ β_1_ φ_b_ (1) φ_a_ (2))/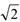. (7)

On this ϕ_a_ and ϕ_b_ stand for the atomic orbitals of the two colliding atoms and α and β for the spin functions.

The corresponding time-dependent Schrödinger equation supplies the following equation:
ψ_±_ (t) = ((ψ_+_ exp (−i
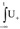
(τ)dτ))−(ψ_-_ exp (−i
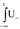
(τ)dτ)))/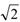
(8)
where U_+_, U_−_: energy of the symmetric and antisymmetric states and ψ_±_: symmetric and antisymmetric wave function, which in case of large distances can be written as:
ψ_±_ = ((α_1_ β_2_+ α_2_ β_1_) ( φ_a_ (1) φ_b_ (2) ± φ_a_ (2) φ_b_ (1))/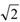
(9)

Formally the spin exchange and resonance charge transfer coincide [32].

### 2.6. The Magnetic Isotope Effect

Isotope effects, as they are usually observed on chemically kinetic examinations, are linked to changes in the nuclear mass (and, thus, to the molecular mass), to changes of the moments of inertias, and on shifts of the oscillation frequency. The theory of kinetic isotope effects [1] yields in some cases quantitative descriptions of averaged energies of oscillation of molecules and activated complexes. In contrast to this the magnetic isotope effect is based on differences in the magnetic properties of isotopes which cause the change of the singlet-triplet evolution in radical pairs.

Usually the hyperfine energies of different isotope nuclei differ (see Section 2.2.1.1.) and, thus, there are different efficiencies for recombinations of radical pairs, which contain these isotopes. E.g., the hyperfine coupling constants of ^1^H and ^2^H differ by factor 6.514 (see [3]). The following will be favourable combinations for isotope pairs for the observation of this isotope effect: ^1^H – ^2^H, ^12^C – ^13^C, ^16^O − ^17^O. Moreover, in the cases of ^12^C and ^16^O it is advantageous that these isotopes do not have nuclear magnetic moments and hence the paramagnetic resonance spectra do not superimpose.

According to [30] there are four remarkable findings, which make the difference between the magnetic isotope effect and the common kinetic isotope effect, which results due to differences in the mass of the isotopes [1]:

(i) Contrary to the classical isotope effect the magnetic isotope effect only occurs in reactions which involve the formation or recombination of radical pairs.

(ii) The magnetic isotope effect may achieve the dimension of some per cent, even for the heavy nuclei ^13^C, ^15^N, ^17^C, *etc*., while the classical kinetic effect is usually smaller.

(iii) The classical effect does not depend on the outer magnetic field, while the magnetic effect shows a clear dependency on 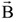. It decreases with increasing 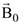. In this case the triplet states [3,27] T_+_ and T_−_ of the singulet-triplet evolution are separated and only the S-T_0_ channel is active. At lower fields all three channels contribute.

(iv) The magnetic isotope effect depends on the hyperfine energy, the spin, the magnetic moments of the nuclei, and parameters which determine the molecular radical pair dynamics, such as the solvent viscosity, radical diffusion coefficients, *etc*. Beside the named kinetic-mechanistic examinations it will be possible to separate the isotopes and to determine the magnetic moments of nuclei in excited states.

### 2.7. Tunnel Effect Phenomenon and Its Relation to ESR and to Isotope Substitution

**Figure 4:**
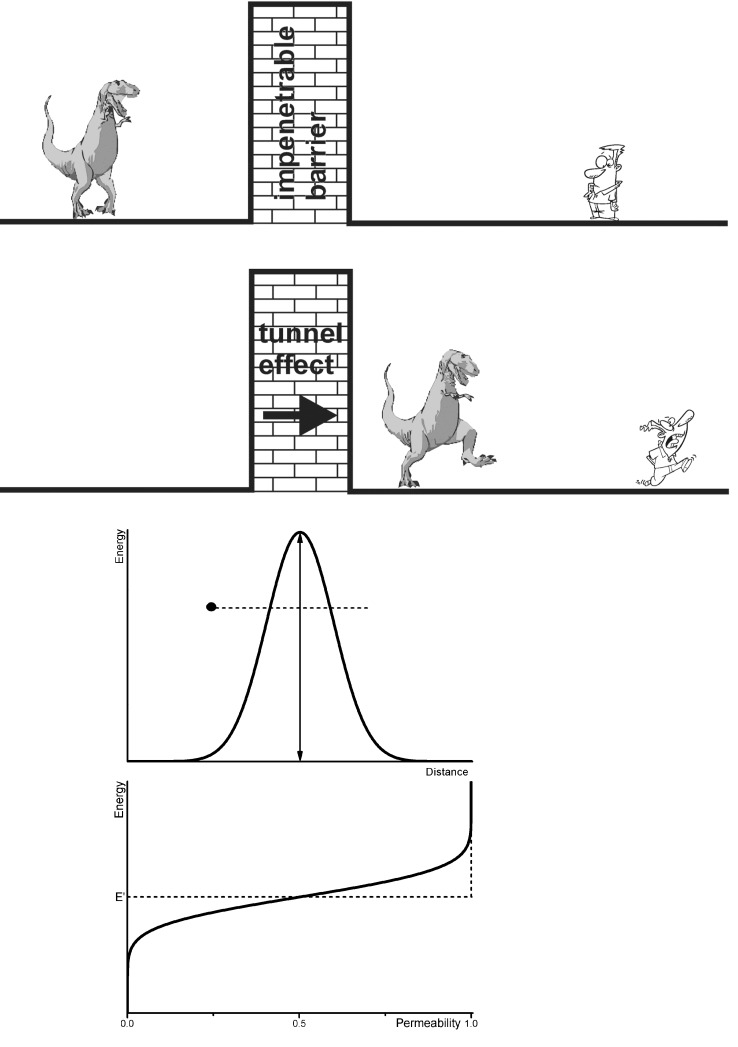
Allegory of the tunnel effect (according to an idea by J. H. Van Vleck cited in [38]) with a classical barrier in Gauss form (particle (•) does not have sufficient energy to get over the barrier) and the probability for tunnelling the barrier (at E’ the probability is 0.5; the dotted line represents the classical course; see also [33]).

The passage of a particle through a potential barrier can occur even if the kinetic energy of the particle is smaller than the height of the barrier. The possibility of a tunnel event decreases rapidly with increasing width of the barrier and decreasing energy of the particle. It is a typical wave phenomenon [32,33,45], whose explanation is delivered by quantum mechanics: The particle (•) “tunnels” the barrier (Figure 4).

The tunnel transfer of an electron can be described as follows [32]:

A^−^ + B ↔ A + B^−^ (10)

where A: Donator, B: Acceptor, and its respective state (wave) function ψ is:
ψ
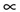
exp (−γ r)(11)
where γ: coefficient of the decay of ψ with distance r

The probability W of the tunnel effect in a unit of time is proportional to ψ^2^ at the donator near the acceptor:

W(r) = ν exp (−γ r) (12)

ν: frequency factor

It is interesting that in general the frequency factor ν does not depend on the temperature and has a dimension of the movement of the electron of about 10^16^ s^−1^ [32]. The parameter γ is associated to the ionization potential I_p_ of the donator:

γ^2^ = 2 mIp/ћ^2^ (13)

m: mass

If an electron transfer is considered, it proceeds from one discrete state of the donator to a discrete state of the acceptor. This requires changes in the coordinates of the nuclei, which again determine the energies of the discrete states of the electrons. *i.e.*, the frequency factor ν does not characterize the movements of the electrons but those of the ***nuclei***. It is a function of characteristic properties of donator and acceptor, and the medium (matrix) as well. The tunnel effect is not limited to changes of the electron state. It appears at the substitution of isotopes, too. The largest effect will occur in the sequence Mu → ^1^H, ^1^H → ^2^H, *etc*. (Mu: {μ^+^e^−^}, the “light hydrogen atom” (see [8,86,87]). As expected the influence of the isotope substitution decreases with increasing atomic number. 

The tunnel effect is not only of importance on the decay of radioactive atoms, but for the kinetics of low-temperature reactions with the transfer of atoms [88], e.g., of H^+^, H•, and H^−^, respectively, for biologically relevant processes [1], for the field of spectroscopy, for processes at electrodes, solid state phenomena, for the visualization of small and smallest particles (e.g., tunnel microscopy).

The temperature dependence is another characteristic of the tunnel effect. While at elevated temperatures nearly Arrhenius behaviour results, at lower temperatures quantum effects dominate the movement of the nuclei and the temperature dependency decreases (double minima potentials). The NH_3_ molecule is an example for this. The double minimum potential is indicated by two equilibrium configurations. And the effect of tunnelling is manifested by doubling every energy level into two levels with a difference according to the tunnel frequency.

Some the empiric criteria for the ‘proof’ of the tunnel effect should be named. According to Bell [33] and LeRoy [89] the following could be stated:

(i) The velocity of a reaction (process) is (mostly) larger than it is predicted by a semi-classical kinetic treatment.

(ii) Deviations from the validity of the simple Arrhenius equation are detected, in which the energy of activation decreases with decreasing temperature and at sufficiently low temperature it becomes independent from temperature (e.g., giving strongly bent curves in the Arrhenius plot). Even at smaller effects the Arrhenius parameters will be smaller as predicted by the semi-classical methods.

(iii) Isotope effects can be anomalously large as well as comparably small [90,91,92].

E.g., the activation energy ΔE_a_ for the hydrogen abstraction by •CH_3_ in CH_3_CN after γ irradiation at 77 K will be 1.4 kcal, while it is 10.1 kcal in the gas phase at 300 K. The rate for the deuterium abstraction is extremely low for low temperature. The ratio k_H_/k_D_ at 77 K is about 20 times larger than in the absence of the tunnel effect.

ESR is one of the most favourite techniques for the detection of tunnel effects [32,33]. E.g., isomeric radicals show clearly different ESR spectra even if they have ‘identical’ energies. The requirement, that the characteristic time t of the technique for measurement must be small compared to 1/ν_t_ (ν_t_: tunnelling frequency), can be fulfilled by the relative wide time window of ESR [3,14,15]. Via a tunnel correction Q value the contribution for the tunnel effect can be quantified [33]. For 1 < Q < 1.1 it can be neglected. For the range 1.1 < Q < 4 a correction is typical for chemical reactions at room temperature. There are quantitative changes only, no new phenomena.

There are nearly no chemical reactions at room temperature with large tunnel effects. At low temperature Q might have values over several orders of magnitude and the examined system might behave completely different (see also [93]).

### 2.8. The Jahn-Teller Effect (JTE)

Jahn and Teller [25,31] stated on the basis of group-theoretical considerations in 1937 that a non-linear molecule with a degenerated electronic ground state is instable. It changes its configuration in such a way that the state of degeneration will be eliminated (Exceptions: Linear molecules and molecules with Kramers’ degeneracy; see ions in crystals).

Therefore the lifting the degeneracy electron states of a paramagnetic centre in a matrix by distortion of the surrounding lattice is called JTE. At sufficiently strong interactions between the centre and the lattice the surrounding lattice atoms can change their states of equilibrium in such way, that a decrease of the local symmetry results, causing the lifting of the state of degeneration (static JTE). The energy content of the system is lower than in the undistorted state. At weaker interactions the gain in energy by distortion is comparable to the zero-point energy of the lattice vibrations and therefore no stable static distortion next to the centre will be formed. Then the state of degeneration will be terminated by the interaction with phonons (dynamic JTE). Due to that, e.g., interesting averaging effects of ESR parameters as a function of the temperature of the solid occur.

### 2.9. Zero-Point Vibrations and Isotope Effects

The zero-point energy of a system is the energy at absolute zero, *i.e.*, the ground state of a system. With respect to the Heisenberg uncertainty principle [45] the kinetic energy of a system does not disappear at absolute zero. Already the (quantum mechanical) harmonic oscillator (e.g., one dimensional) supplies a frequency spectrum with the energy.

E_n_ = h ν (n + 1/2) (14)

n: 0, 1, 2 ….

With this the ground state has the energy:

E_0_ = h ν/2 (15)

At T = 0 K the movement of the particles cannot “freeze completely”, due to the fact that the location would be fixed then (Δq = 0) and the impulse p would be zero (Δp = 0; Δq Δp ≥ h/2).

**Figure 5:**
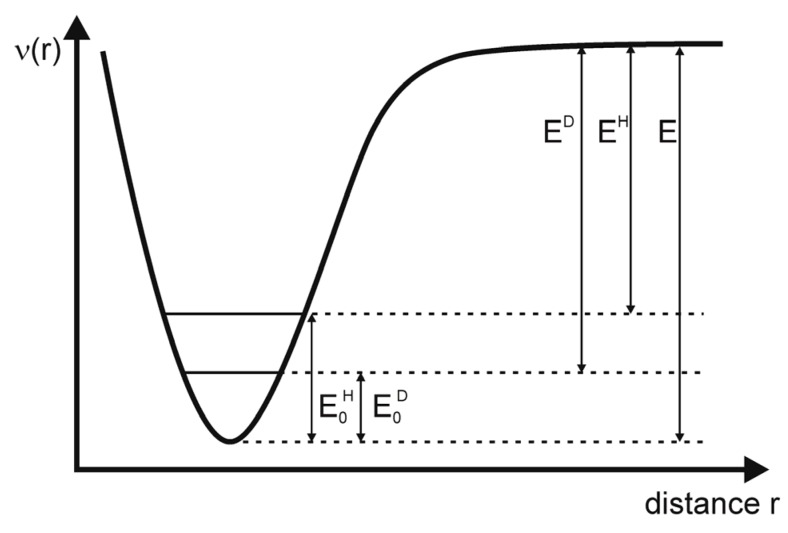
Influence of the zero-point energy on the dissociation of R-H and R-D fragments (E_0_^H^ and E_0_^D^: zero-point energies of hydrogen and deuterium; adapted from [33]).

Zero-point energy effects are responsible for finite rates of reaction at temperatures close to absolute zero. This is true e.g., for hydrogen abstraction and others, whereas there are predictions for large ratios of k_H_/k_D_ (see e.g., [93]. The phenomenon of zero-point energy and its effect on processes of dissociation in chemistry can be demonstrated easily by means of the dissociation of X-H and X-D fragments. The force constants kdo not differ significantly for both isotopes (
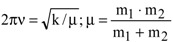
), but the reduced mass is smaller for the X-H than for the X-D fragment and consequently the oscillation frequencies ν_H_ > ν_D_ (in reality the ratio ν_H_/ν_D_ is about 0.7) and the zero-point energies behave as E_0_^H^ > E_0_^D^ [33]. Thus, the dissociation energies are E^H^ > E^D^ (see Figure 5) and the dissociation of the hydrogen component will be faster (there is a smaller activation energy). This is, among others, manifested in the higher pH value of D_2_O in comparison to H_2_O at the same temperature.

### 2.10. Parameters Determining the Habit of the ESR Spectra (ESR Response)

(i) parameters which influence the shape of experimental ESR spectra or which influence the ESR response S: 

instrumental parameters with direct relation to the resonance effect:
S = S (ν_MW_, ν_RF_, P_M_, P_RF_, 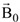, t_i_, Grad B_i_, 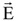, …)(16)
where ν_MW_: microwave frequency (⊥ or ||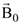); ν_RF_: radio frequency; P_M_, P_RF_: microwave or radio frequency power; 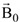: outer ‘static’ magnetic induction; t_i_: different time functions for the variation of the other parameters (cw and impulse techniques); Grad B_i_: gradient of one component of 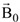, e.g., for recording spatially dissolved spectra (ESR tomography); 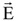: electric field e.g., for interaction with quadrupole moments.

(ii) parameters which comprise the changes of the structure of paramagnetic species including the interaction within the paramagnetic unit: anisotropies of the spin coupling matrices, exchange interactions (particularly spin exchange parameters as well as exchange interactions within a species at “fixed” geometry, *i.e.*, mainly the parameters of the spin-Hamilton operator (see [18,22]))(remark: the commonly used spin-Hamilton operator is independent of time and mass; thus isotope effects mainly result in the number of lines and intensity of the hfs as well as on the value of the hyperfine coupling).

(iii) parameters which determine the change of the macroscopic state and the structure of the complete sample. In principle there many different kinds of influences which have to considered and which act upon the thermal, mechanical, electrical, optical, magnetic, and chemical properties of substances and their coupling with the spin properties (*i.e.*, it is related to the coupling of the electron spins with all accessible degrees of freedom of the recent sample).

(a) preparation of the macroscopic state of the sample by variation of T, p, v, 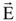, 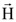, c_i_, …(c_i_: concentration of components which, e.g., determine the pH value or the concentration of educts or products of chemical reactions); 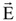 is responsible for polarizations or chemical reactions (e.g., electrode reactions; see below); the state of magnetization of the sample may by varied by 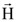 of different origins.

(b) preparation of the microscopic state of the sample by changing the electron configuration of the atoms or molecules, respectively, by redox reactions, optical excitation or ionizing radiation (among others there may be internal changes in concentration; it cannot be assumed that the nuclei are “fixed” in all cases, but the respective movements have not to be considered in any case), respectively.

(c) dynamics of paramagnetic centres: parameters of the dynamics of the complete paramagnetic centre, *i.e.*, diffusion, reorientation, rotation of molecular fragments, collisions, and spin exchange, isotope effects in the matrix (isotopic dilution, zero-point energy effects, *etc*.; even here the atomic nuclei can be considered as mainly “fixed” in the centres)

(d) parameters of the phenomena which are caused or modified by the *movement of nuclei* in the paramagnetic species: vibronic couplings, zero-point vibrations, dynamic Jahn-Teller effects, (tunnel effects) … and the respective isotope effects via the mass of the nuclei or anharmonic contributions to the interaction potentials.

**Table 2:** Matrix arrangement of the spin (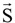,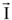), orbital (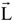), and quadrupole moments Q ^††^, as well as electric (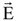), and magnetic fields (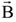) ^‡‡^ for symbolizing the spin couplings (with “fixed” atomic nuclei ^§§^).

	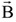	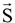	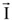	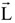	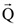	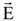
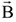	I	II	II	IV	V	•
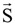		VI	VII	VIII	•	•
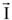			IX	X	•	•
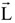				•	•	•
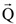					•	XI
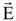						***

^††^ nuclear quadrupole moments which interact with electric and magnetic fields; ^‡‡^ in form of the magnetic induction; ^§§^ • stands for small terms (see [87,94] within the total Hamilton operator, which are time and mass independent and do not contribute much to the ESR response; I: diamagnetism 1^st^ and 2^nd^ order of perturbation theory; II: Zeeman term H_Z_ with coupling parameter ĝ_e_; III: nuclear Zeeman term H_N_ with coupling parameter ĝ_N_; IV: Zeeman term H_L_ with coupling parameter ĝ_L_; V: nuclear quadrupole interaction with 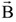; VI: spin-spin interaction H_SS_ with coupling parameter 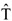 (ESR fine structure) or J, respectively; VII: ESR hyperfine interactions, Fermi-contact interactions, dipolar hyperfine splitting; VIII: spin-orbit coupling with parameter λ; IX: nuclear spin-nuclear spin interaction J_ij;_ X: nuclear spin-orbit interaction; XI: nuclear quadrupole-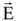 interaction; *** these interactions will not be considered and explained.

### 2.11. Models for the Parameterization of ESR Spectra; Phenomenological ESR Parameters

Phenomenology of the ESR parameters

(i) “nearly model free” parameters (*i.e*., general spectrometric parameters) such as position of lines (often given in effective g factors 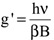 or 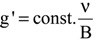), line form (Gauss, Lorentz, and Voigt profiles) as well as line widths.

(ii) parameters of spin depending but time and mass independent interactions. Similarly, the concept of the spin-Hamilton operator Ĥ_spin_ will be in the central position [3,22,82] including the introduction of statistical distributions of the coupling parameters [89,90]. The spin-Hamiltonian Ĥ_spin_ represent the disturbance energy of a system as a function of the spin operators. Individual terms of Ĥ are shown in Table 2 and e.g., in [3,22]. A more general representation of the relations between the properties of isotopes and the ESR response is given in Figure 1.

(iii) models for determining time and mass, as well as spin depending effects such as: dynamic models or equations of motions, respectively, for describing the molecular reorientation [10,95], diffusion, spin exchange [35,96], tunnel effects [32,33], vibronic couplings, the dynamic Jahn-Teller effect [25], chemical reactions, spin relaxation [97] with the parameters T_1_ and T_2_ [3,98], and spin-phonon interactions [4,83,85,99].

(iv) quantum mechanical models for the calculation of energies, state functions, electron and spin densities [100,101,102], calculations of the spin-Hamilton operator under consideration of perturbation terms, solution of the time-independent Schrödinger equation considering isotope effects.

## 3. Selected Examples

### 3.1. Determination of Concentrations of Paramagnetic Particles in a Sample by Means of ESR

On finding parameters for experimental ESR spectra the first question which arises is that for the number of spins and their relevance in the system under investigation (similarly to all other fields of spectroscopy). That question can be answered nearly without using theoretical models [16,75,103,104,105,106]. In other words, ESR can be used for the purpose of quantification. Nevertheless the determination of concentrations is challenging. In ESR spectroscopy generally the first derivative of the signal is registered and the signal area is proportional the number of spins. Thus, the double integration of the spectrum is needed for the determination which easily gives large errors [16,75,103,105,106]. Considering the most important sources of errors, one can determine the concentration of paramagnetic species e.g., as a function of time or other suitable spectral parameters. For this it is important that the spin properties of the particles itself are used. In this way it will be possible to observe kinetic isotope effects. As an example the examinations of the reaction of H or D, respectively, with CH_4_ by Kurylo *et al*. [107] shall be used. For the reactions:

H• + CH_4_Ý H2 + •CH_3_ (17)

and:

D• + CH_4_Ý HD + •CH_3_ (18)

the constants k_1_ and k_2_ have been determined to be k_1_ = 6.25 10^13^ exp [−11600 ± 150/RT] and k_2_ = 4.5 10^13^ exp [−11100 ± 150/RT], *i.e*., in the expected relation for the isotopes.

Using the much larger isotope effect of muonium [8,108] Roduner could obtain further information [109]: The competing effects zero-point energy and tunnel effect could be separated from the kinetic isotope effect as follows: if k_Mu_/k_H_ < 1 the zero-point energy dominates in the transition state, and k_Mu_/k_H_ > 1 for the domination of the tunnel effect.

### 3.2. Influence of the H-D Substitution on the g Factor

Coupling of the electron spins with the surrounding magnetic fields will be realized by the ĝ tensor (g coupling matrix). Next to the outer magnetic induction 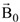 the contributions of the orbital momentum has to be considered. The influence of the nuclear masses of the involved atoms is very small, thus there are only very few investigations with respect to the influence of isotopes in that field.

Walther *et al*. [110] was successful in measuring the ratio g(H)/g(D) = G for the 1s states with an accuracy of 3 10^−11^ by means of hydrogen maser. With it the ratio (G – 1) could be determined to be 7.221 10^−9^ (see also [111]). For the g factor of ^1^H• a value of 2.0022838 is given by Weil and Bolton [3] (see also [112,113]).

The vibronic reduction factor should be applicable for all physical ground state properties of electronic origin and thus have an influence on the orbital contribution of g, *i.e*., g_L_ (see [25]).

### 3.3. Selected Hyperfine Effects

#### 3.3.1. ESR Detection of Effect of Isotopes in Natural Abundance

**Figure 6:**
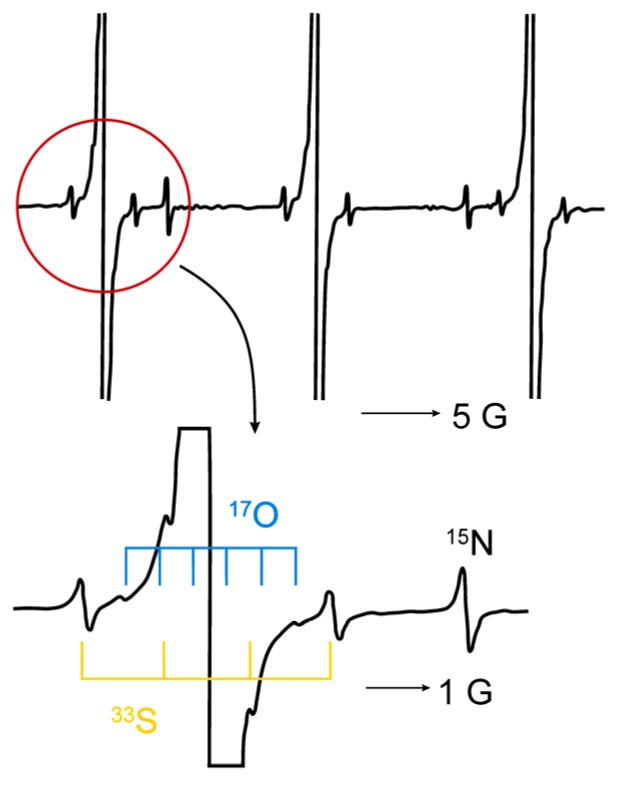
ESR hyperfine structure of K_4_[(SO_3_)_2_NO]_2_ in a system of reversed micelles including the respective isotope splittings (inset) using the natural abundance of the isotopes (adapted from [38]).

An impressive demonstration of the ability of the simple cw ESR is supplied by Okazaki [114], with respect to the detection of isotopes in natural abundance (see also [20,115,116]). Solutions of K_4_[(SO_3_)_2_NO]_2_ (PADS) have been measured in a system of reversed micelles [117] in order to increase the sensitivity and the spectral resolution. With that it was possible to detect beside the well-known ^14^N hfs triplet (see Figure 6) the hfs of ^17^O (in SO_3_; 3.7 × 10^−2^% natural occurrence; I = 5/2; 6 lines with 0.537 ± 0.1 G hfs splitting), of ^33^S (0.74%; I = 3/2; 4 lines with 1.282 ± 0.1 G hfs splitting), and of ^15^N (0.365%; I = 1/2; 2 lines with 18.35 ± 0.056 G hfs splitting) could be observed (the ^17^O splitting in the NO fragment was 20.55 ± 0.26 G [116]). Altogether the authors were successful in assigning the spectral contributions to the overall spectrum. Moreover they succeeded to determine the different spin densities at ^17^O in the NO and SO_3_ fragments. The sign of the ^17^O hyperfine splitting in Fremy’s salt was determined by Luz *et al*. [11].

A specific aspect of the hyperfine interaction shall be mentioned here with respect to the application of the isotope ^99^Tc in radio-pharmacy. ESR supplied much information for refining the structure of ^99^Tc coordination compounds, as well as the chemical knowledge about that isotope [12,13].

#### 3.3.2. Evidence for Trapped Muonium, Hydrogen, and Deuterium Atoms at Ambient Temperature

**Figure 7:**
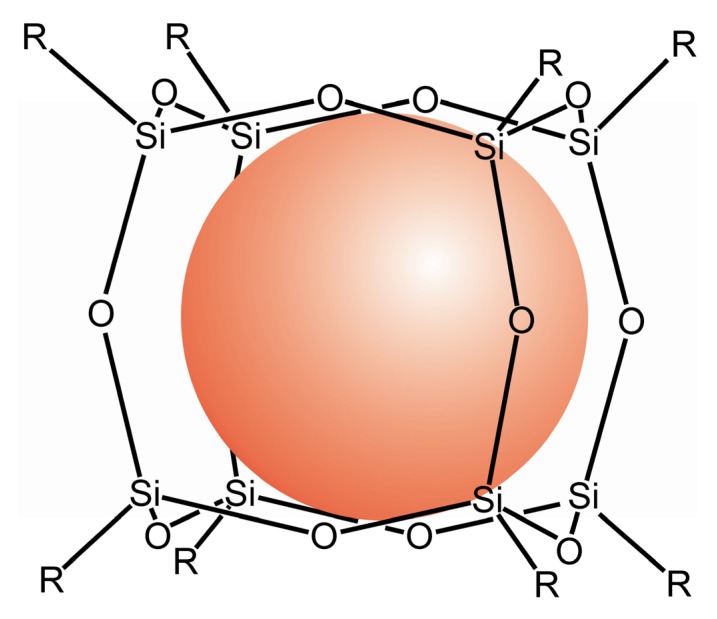
Model of a Si_8_O_12_R_8_ cage with incorporated H• (van der Waals radius).

**Figure 8:**
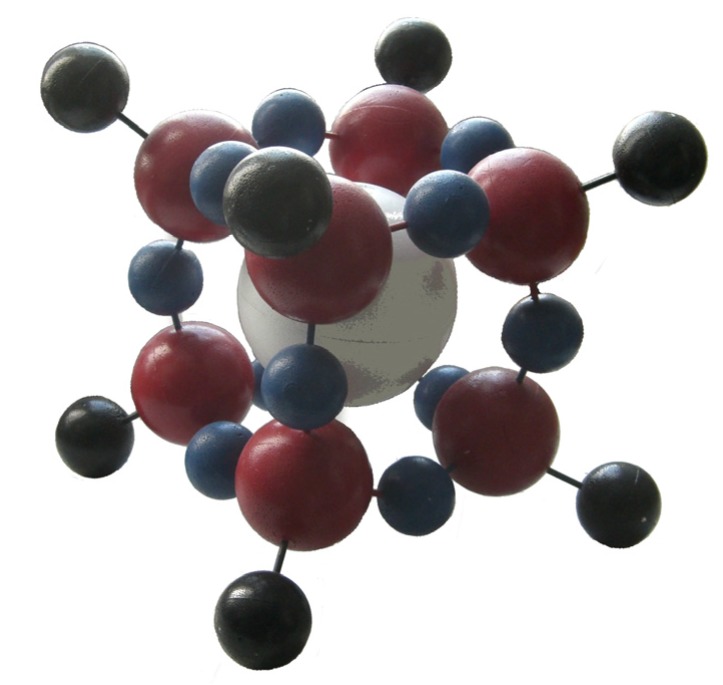
Photograph of a Si_8_O_12_R_8_:^1^H model.

Compounds with the structural unit Si_8_O_12_ (see Figure 7, Figure 8) have been proven to be useful for trapping reactive species like ^1^H•, ^2^H•, and Mu (μ^+^e^−^ [86,87,118,119]) and to stabilize them for longer periods of time. These systems are not only interesting objects for investigations of the properties of the named paramagnetic species but they are useful as probes in their surrounding (e.g., for the detection and quantification of ^3^O_2_). Due to steric reasons of the cage there will be no real chemical reaction between H• and ^3^O_2_ but via the spin exchange the concentration of ^3^O_2_ can be determined as well as the frequency of non-reactive collisions in solution and solids.

**Figure 9:**
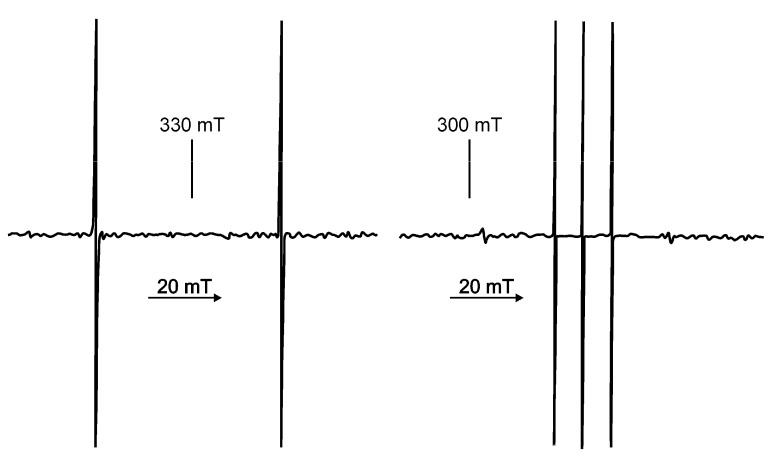
ESR spectra of H• (I = 1/2) and D• (I = 1) in solid Si_8_O_12_R_8_ matrices.

**Figure 10:**
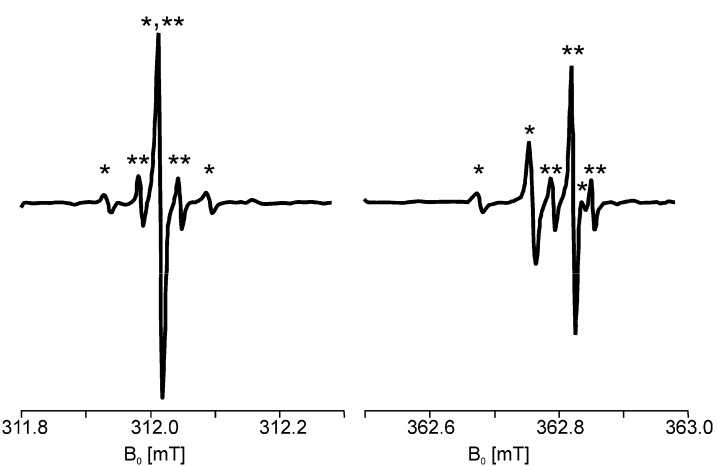
ESR spectra of degassed solutions of γ irradiated Si_8_O_12_R_8_ cages in C_2_Cl_4_ at 293 K. *: H• in the cage with R = propyl **: H• in the cage with R = methyl.

Figure 9, Figure 10 show the atomic hydrogen trapped in the Si_8_O_12_ cage which is symbolized by its van der Waals radius. The respective ESR spectrum for the solid compound is displayed in Figure 9. As expected the trapped ^2^H• gives a 3-line spectrum with the reduced hyperfine coupling constant (a = 7.8) due the smaller gyromagnetic ratio γ_H_/γ_D_ = 6.51 and the nuclear spin I(^2^H) = 1. The proof for the location of the hydrogen atom in the cage can be seen in Figure 10. The cages Q_8_M_8_ and EtT_8_ [120,121,122] had been irradiated with γ-rays. The line widths in the degassed solution are in the range of 2 – 9 μT. The symbol * indicates the signals of EtT_8_:^1^H and ** stands for Q_8_M_8_:^1^H. The satellites to the ^1^H doublet are a result of the interactions with the ^29^Si atoms of the cages. It has to be considered that the number of ^29^Si (I = 1/2) atoms per cage is determined by the natural abundance of ^29^Si (4.7%) and the probabilities of the occurrence in the cages (1 times ^29^Si, 2 times ^29^Si, *etc*.). The calculated probabilities and, thus, the percentage part of the ESR intensity correspond well with the experimental findings. Altogether this is the *proof* for the trapping of the hydrogen atom inside the cage.

**Figure 11:**
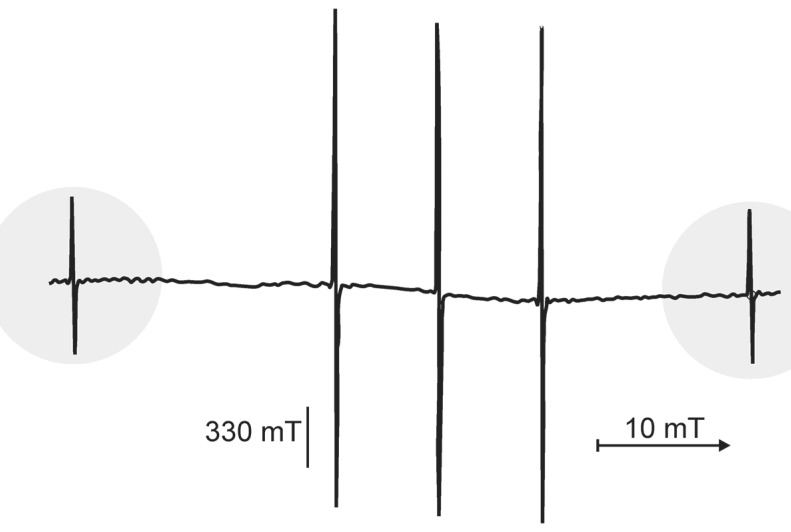
ESR spectrum of a γ irradiated Si_8_O_12_R_8_ cage dissolved in C_6_D_12_ at 300 K (in the centre the line triplet of the D• atoms; at the outside the doublet of H•).

Figure 11 reports about the origin of the ^1^H• and ^2^H•, respectively. EtT_8_ was irradiated at 300 K dissolved in C_6_D_12_. About 82% of the ESR signal area originate from ^2^H•, while about 18% are related to ^1^H•. That means, intermolecular processes take part in the trapping. Only about 18% of the ^1^H• can have their origin from the CH groups of the cage itself.

The cages proved to be suitable not only for the trapping of ^1^H and ^2^H. The light hydrogen isotope muonium could also be trapped inside these cages at room temperature [86]. The muonium is the ultra-light isotope of hydrogen [87,109]. Under physical aspects μ^+^ is considered to be a heavy electron and in chemistry a light proton. Its mass is only 1/9 of that of hydrogen and it can be incorporated in different systems. Except the mass effect the μ behaves like a hydrogen isotope. It is the most sensitive probe for all kinds of mass dependencies, like effects of zero-point energy and tunnel effects. E.g., in EtT_8_ a hyperfine coupling constant of muonium of 4405.9 MHz compared to 1415.58 MHz for EtT_8_:^1^H, and 217.314 MHz for EtT_8_:D can be observed. Altogether, the absolute values of the hyperfine coupling constants compared to the values of the isotopes in vacuum clearly show the interaction of these atoms with their surrounding; they act as spin probes (see [123,124,125]). 

### 3.4. Selected zfs Effects

Isotope effects in the ESR fine structure of Mn^2+^ in Ca(OH)_2_ and Ca(OD)_2_ have been examined by Holuj and Kwan [126]. At low temperatures there are relatively large differences with respect to the zfs parameter ${ƅ}_{0}^{2}$. ${ƅ}_{0}^{2}$ symbolizes a spin coupling parameter of second order in the spin-Hamilton operator [see [22,82] and Equation (5)] and it can be interpreted as a measure for stretching or compressing of a Mn^2+^ coordination polyhedron. The analysis of the ESR parameters concerning the lattices vibrations revealed, that the hyperfine coupling constant of ^55^Mn changes only slightly over a large temperature range (about 4.3%), but the fine structure parameter ${ƅ}_{0}^{2}$ changes even its sign. Next to the thermal expansion of the sample with increasing temperature a modulation of the crystal electric field by the lattice vibrations occurs. The development of that field in terms of displacement of the participating atoms including orbital lattice interactions showed, that the contributions of the involved acoustic modes to the modulation process are reciprocally proportional to the masses of ^1^H and D [126].

For these findings it is interesting to watch the subtle changes of the fine structure by isotopic marking, but that the changes appear via changes in the vibrational modes of the matrix.

The influence of isotopes on the fine structure of ESR spectra is described in [127,128,129,130]. The role of the spin-phonon coupling was investigated by Böttger [99] on examining the line splitting of the ESR spectrum of Ni^2+^ in MgO, while Tomioka *et al*. [131] studied steric and isotope effects in triplet carbene systems.

### 3.5. Examples for the Jahn-Teller Effect and Vibronic Couplings

The ESR detection of the static Jahn-Teller distortion in cationic radicals with originally degenerated occupied molecular states has been done already with several examples such as SF_6_^+^ [132], benzene^+^ [133,134], *etc*. At higher temperatures the hyperfine structure for the benzene cation results in six equivalent protons due to the fast exchange of the three equivalent distorted structures [135]. This phenomenon is called pseudo-rotation. The influence of the isotopic substitution on the Jahn-Teller distortion was demonstrated by means of cations of methane, cyclopropane, and cyclohexane [134]. The respective theoretical background can found in [136]. Partially deuterated benzene cation radicals are the objects in the examinations of Toriyama *et al*. [134] while Iwasaki *et al*. [137] examined ethane radicals in SF_6_. It was found that the kind of distortion of the cations is unique with respect to the sites of the D substitution in all examined matrices. For the ground states D_2h_ symmetry results and the remaining C-H bonds are localized in the distorted structure at sites with higher spin density.

In the simplest case the zero-point energy for the stretching vibrations of C-H is proportional to (k/m_H_)^1/2^ (k: force constant). On exchanging H and D the complete zero-point energy depends on the position in the cation. The criterion for that consists in the strongest decrease of the zero-point energy in case the D atom occupies a position with a larger force constant. That can be realized with a shorter bonding and smaller spin density.

The addition of muonium to benzene gave an interesting effect as described by Eckert *et al*. [87]. This reaction was about twenty times faster than that of ^1^H and the activation energy was about two third smaller [138]. That finding will be caused by a dominance of the tunnel effect which will superimpose that of the zero-point energy in the transition state.

### 3.6. Examples for the Tunnel Effect

For the first time Zamaraev and Khairutdinov [139] could provide the experimental proof that the reactants have an unusual large distance from each other, *i.e*., about 1.4 Å, in the moment of reaction. This was found for the reaction:

e_tr_^−^ + O^−^ → O^2−^ (16)

Both reactants are ESR active and can be observed separately because of the different spin-orbit coupling contributions to the ĝ tensor. Moreover, from the line widths of the signals the distance between the paramagnetic species can be determined. Further on there is a possibility to determine the actual concentration of the paramagnetic reactants and products, respectively. It turned out that in the range of 4.2–93 K the main reaction channel appears to be a non-activated tunnel process for the electron transfer, which changed to an activated one at higher temperatures.

Due to the fact that ESR spectra sensitively reflect changes in structure and the local environment of the paramagnetic species, isomerizations, interconversions of radical pairs (see [30]) and isolated radicals, respectively, as well as transformations of diradical pairs in crystals can be observed. Examples for this are described by Bell [33] and Williams *et al*. [140,141,142,143]. E.g., :

CH_3_ + CH_3_X Ý CH_4_ + •CH_2_X (X = CN, NC, OH) (19)

CH_3_ + CH_3_OD Ý CH_4_ + •CH2OD (20)

Due to the usage of CH_3_OD the line widths was reduced caused by the smaller magnetic moments of the D nuclei.

Another example for the influence of isotopes on the tunnel effect will be the isomerisation of 2,4,6-tributylphenol into 3,5-di-*t*-butylneophyl [144] which was carried out in comparison to the completely deuterated species. In the range from 28–200 K a strong change in activation energy took place indicating a distinctive isotope effect. For that the ratios of the rates k_H_/k_D_ have been determined which particularly at low temperatures are much above the predicted one for the model of the absolute reaction rate. Thus, one can state that the complete process is dominated by the tunnel effect.

The tunnel effect of H atoms could observed on changing radical pairs in γ irradiated crystalline dimethylglyoxime, too. The vanishing activation energy in the range of 4.2–50 K is also a sign for a tunnel process [32,145,146]. The respective ^1^H/^2^H isotope effects have been examined by Jakimtschenko and Lebedev [145]. It could be shown that the conversion of radical pairs in the temperature range 77–310 K occurs via intra and intermolecular migration of bonds. The isotope effects are pronounced particularly at low temperatures. The basic mechanisms are mainly determined by hydrogen tunnelling.

Another interesting isotope effect at the para-elastic relaxation has been shown by Pfister and Känzig [147] by means of ESR. The paramagnetic species ^16^O_2_^−^ and ^18^O_2_^−^ have been examined in KCl, KBr, and KJ at temperatures of 1.3–4.5 K under uniaxial pressure. The observation of the elastic relaxation of the systems after pressure release was done via recording the ESR intensity as a function of the time. Under a molecular aspect the relaxation was expressed by the turning of a molecule from one equilibrium orientation to another one due to undoing the distortion after the pressure release. Despite the ‘jump rate’ of the paramagnetic molecules had been very small at the selected temperatures the sensitivity of ESR allowed the observation of the time-depending course via changes of the line intensity of the ESR signals. At T < 4 K the rate of reorientation was directly proportional to the temperature. On turning the molecules the present potential barriers have been tunnelled successfully. On that a relatively large isotope effect of the rates of reorientation for ^16^O_2_^−^ and ^18^O_2_^−^ could be detected. It is interesting to state that the differentiation between the species ^16^O_2_^−^ and ^18^O_2_^−^ (both have I = 0) by classical ESR parameters is not possible.

### 3.7. Influence of the Zero-Point Vibrations

The influence of the zero-point vibrations on the superhyperfine interactions of ^1^H• and ^2^H• in KCl could be shown by Spaeth [148] by means of ESR. The isotropic superhyperfine splitting constants had been smaller in case of ^2^H•. The ratio of the couplings with the potassium nuclei (I = 3/2 for ^39^K) had been a_H_(K)/a_D_(K) = 1.165 and with chlorine (I = 3/2) 1.026. About 30% of a_H_(K) and about 10% of a_H_(Cl) could ascribed to zero-point vibrations.

The lighter ^1^H• causes a larger energy and amplitude of the zero-point vibration and a stronger influence on the hyperfine interaction. Finally the average effect of the vibrations on the superhyperfine structure was observed. This means an admixture of different core-orbital contributions to the spin density of the ESR active centres takes place. Further results for H and D atoms in alkali halide crystals are given in [149].

The examinations of Morton and Preston [81] supplied an example for the influence of isotopes on the isotropic hyperfine interactions using the F_2_NO radical. The ^19^F coupling increases with the mass of oxygen and nitrogen (^15^N and ^17^O substitution). As expected the original nitrogen coupling changes from a triplet to the ^15^N doublet with a larger splitting.

Hints to quantum-mechanical tunnelling processes of methyl protons at 20 K could be obtained from the analysis of the ESR spectra of the partially deuterated tetramethysilanes [Si(CH_3_)_4_^+^ and others] [150]. By means of the ^1^H-^2^H substitution in radical cations of CH_3_OCH_2_F and CH_3_CCl_3_ and the complete analysis of the ESR spectra, accompanied by *ab initio* DFT-MO calculations, the conformation of the cations could be determined.

The influence of the ^2^H substitution onto the conformation and rotation of the CH_3_ groups in the dimethylether cation could be shown by Itagaki *et al*. [101]. The ^1^H hyperfine constant showed up to 100 K nearly no temperature dependency different to the ^2^H system because of the difference in the zero-point vibrations of the different rotational conformers.

The proof for intermolecular hydrogen bridges in the hydroxysemiquinone provided Gendell *et al*. [151] by means of ^1^H-^2^H isotope effects on the hyperfine structure. Obviously the vibrational effects in a hydrogen bridge play a decisive role. The ratio of the coupling constants a(^1^H)/a(^2^H) was in the range of 7.2−7.8 larger than the expected value of 6.51 out magnetic data.

The reduced mobility of ^2^H has an effect on the values of the hyperfine splitting in isotopically substituted 1-hydroxypyridil radicals [152].

Papers concerning the simulation of ESR spectra considering isotopic substitutions on the hyperfine structure including the respective computer programmes are widespread in the literature (see e.g., [101,153,154]).

### 3.8. Isotope Effects of the Matrix

#### 3.8.1. Stationary Effects

This concerns the detection of the properties of the surrounding of paramagnetic species. In this connection the term matrix should be understood to be the lattice of a solid, as well as solvents, or other fluid systems. Thus the isotopic substitution might be done in: (i) the spin probes as well as in (ii) the units of the matrices. Further differentiation in: (iii) static and (iv) dynamic effects seems to be useful.

##### 3.8.1.1. H-D Exchange and Fermi Contact

The values for the Fermi contact interaction [3] of the hydrogen isotopes in liquid and solid H_2_O are below the vacuum values. They depend on the mass and give a negative temperature coefficient in the liquid phase. It means there is some spin delocalization to solvent molecules. Moreover, there is a strong isotope effect in mixtures of H_2_O and D_2_O [124].

Furthermore there is an indication for the existence of clathrate-like cages in water. Other chemically interesting effects of H-D exchange were observed by Magin and Morse [155] and Pfirrmann *et al*. [156].

##### 3.8.1.2. Spin Probes and Polarity of the Matrix

**Figure 12:**
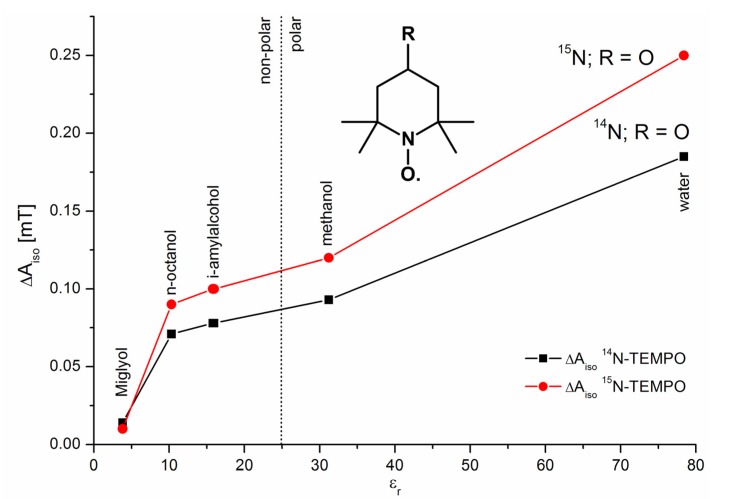
Formal differentiation of the polarity of solvents by means of the values of the hyperfine coupling constants in relation to the respective dielectricity constants ε_R_. The ordinate shows the differences ΔA_iso_ with respect to coupling constant of toluene.

Under practical chemical considerations the term polarity is used in chemical synthesis, e.g., to describe the influence on the course of chemical reactions by selecting and applying suitable solvents. The relative dielectric constant ε_r_ is a possible means to indicate the polarity of a solvent [157]. The hyperfine coupling constants of spin probes, dissolved in the respective solvent are related to polarity [131]. The reason for that is the fact that in polar media the accommodation of the polar constituents of the structure of the spin probes is favoured and the spin density at the nitrogen atom will increase (see Figure 12). With it the hyperfine coupling increases, too. As shown in Figure 12 fluid media can be formally subdivided into polar and non-polar (ε_r_ < 25). In that way the selection of a suitable solvent for specific reaction can be selected by their hyperfine coupling. For the determination of the polarity the usage of ^15^N spin probes is useful, because the larger hyperfine coupling responds more sensitively on changes of ε_r_.

Distance measurements in complex systems have been performed by Jeschke *et al*. [158] using marked nitroxyl spin probes.

The literature provides a large number of papers concerning the simulation of ESR spectra of selected transition metal complexes, as e.g., considering the surrounding matrix of complexes of copper isotopes in their natural abundance [153].

##### 3.8.1.3. Spin trapping

The method is characterized by transferring short-living paramagnetic species, mainly radical intermediates, to longer living radicals by reacting with so-called spin traps (e.g., DMPO). For that, there are similar considerations as stated already [6,7]. Also in these cases the identification of the species by isotopic marking (^1^H → ^2^D, ^14^N → ^15^N) will be easier and the sensitivity will be enhanced [159]. The trapping of the superoxide anion and the identification of the trapped radical species could be improved after applying isotopically marked spin traps as it is shown in [160].

#### 3.8.2. Dynamic Effects

##### 3.8.2.1. Spin Relaxation

As mentioned in Section 2.2. the usage of isotopes is reflected in spin relaxation times T_1_ and T_2_. The findings of Du *et al*. [161] and Biller *et al*. [162] shall serve as examples for that.

In the range of rapid tumbling (0.5–0.1 μs) the relaxation times T_1_ and T_2_ in liquids approach each other (T_1_~T_2_). Biller *et al*. [162] found larger values for T_1_ as well as T_2_ for selected ^15^N spin probes than for the respective ^14^N probes. This and the smaller line widths improve the signal-to-noise ratio, too. All this is based on the differences in the nuclear magnetic moments und the nuclear spin. Beside the thermal modulation of the anisotropic nitrogen hyperfine structure the modulation of the spin-rotation interaction contributes to T_1_.

##### 3.8.2.2. Diffusion of Spin Probes and ESR Tomography

**Figure 13:**
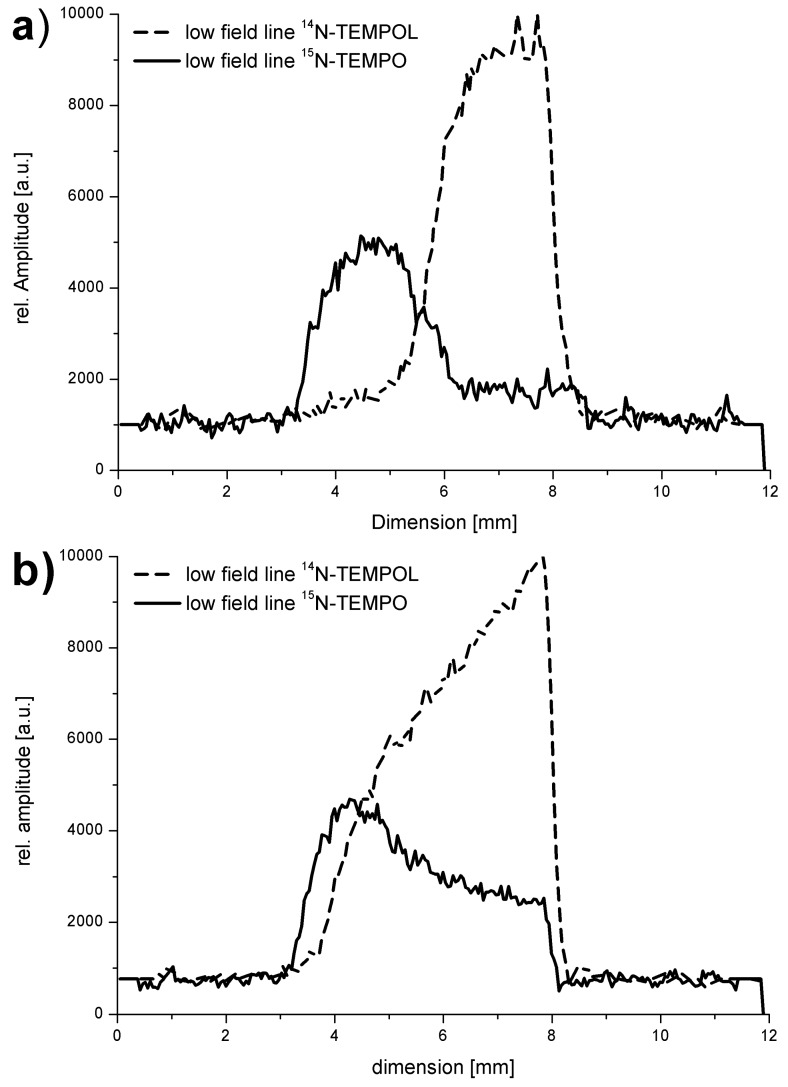
Lateral diffusion of the spin probes ^14^N-TEMPOL and ^15^N-TEMPO dissolved in the ionic liquid BuMImBF_4_ at 300 K. The signal amplitudes are displayed as function of the spatial coordinates.

The isotopic marking of the spin probes ^15^N-2,2,6,6-tetramethylpiperidin-1-oxyl (TEMPO) and 4-hydroxy-2,2,6,6-tetramethylpiperidin-1-oxyl (TEMPOL) allows the observation of their lateral diffusion in opposite directions in a one-phase system by means of ESR tomography [163]. Figure 13 displays the profiles of the signal amplitudes after 10 and 135 min, respectively, for the diffusion of the spin probes in the ionic liquid butylmethylimidazolium tetrafluoroborate (BuMeImBF_4_) [163]. By means of the marking the concentration profiles can be assigned unambiguously and specifically for the respective probe including their spatial distribution.

**Figure 14:**
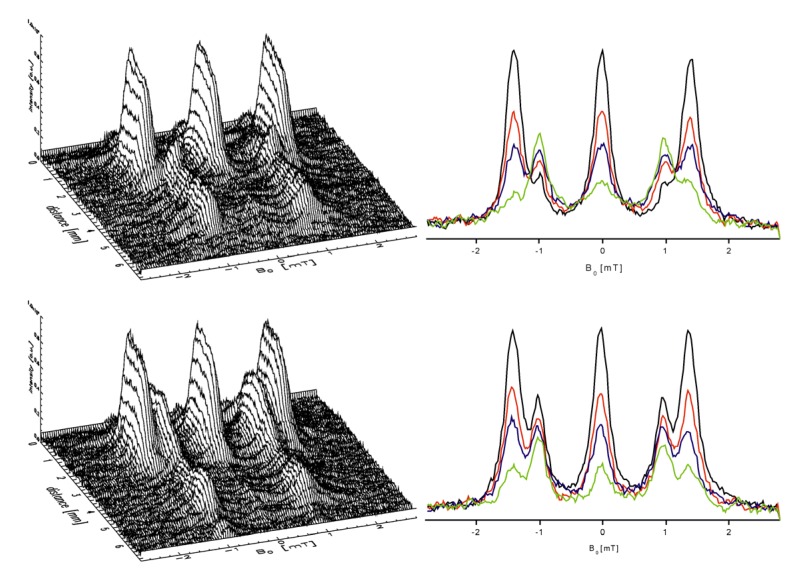
Reverse diffusion of the spin probes ^15^N-TEMPO and ^14^N-TEMPOL in Precirol and Miglyol, respectively, after 5 and 240 min. Spectra-spatial images including of selected ESR spectra in different depths of the two-phase object (black: 2.17 mm depth, red: 2.71 mm depth, blue: 2.85 mm depth, green: 3.25 mm depth).

The lateral diffusion of two spin probes in a two-phase system can be similarly tracked by ^15^N-marking and ESR tomographic examination as shown in Figure 14. For that purpose ^15^N-TEMPO was dissolved in Precirol^®^, while ^14^N-TEMPOL was dissolved in Miglyol^®^ and brought into contact within a capillary tube. The spectral-spatial images and the respective spectra in different depths show the progress of the distribution after 5 and 240 min [37], respectively, are displayed in Figure 14.

Particularly in biological systems, ESR imaging requires the usage of spin probes and conditions for measurements which give acceptable signal-to-noise ratios. Such systems are usually characterized by high dielectric losses due to microwave absorption and it might be generally advisable to perform the examinations at smaller microwave frequencies than to use the standard frequency of about 10 GHz. Another method for improving the signal-to-noise ratio will be the increase of the spin probe concentration. Unfortunately this is not always possible because of the toxicity of the spin probe and, moreover, an increase in line widths, whereas the isotopic substitution of ^1^H by ^2^H will improve the situation because the small nuclear moments of the ^2^H nuclei give smaller magnetic dipole-dipole contributions to the line widths [164]. The ^14^N-^15^N substitution in the nitroxyl spin probes is favourable, too, because to the reduction from three to two lines also improves the signal-to-noise ratio.

##### 3.8.2.3. Spin Exchange and Chemical Reactions

The direct determination of ^3^O_2_ in solution (e.g., in H_2_O) is very difficult due the small distribution coefficient of ^3^O_2_ and the relaxation times of the respective spin states are very short and, thus, the determination of 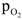, too. Examples for this are the changes of 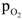 in the course of the Maillard reactions [165], the analysis of water samples, in which 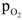
provides environmental information about the sum of further dissolved substances and about activity and state of biosystems.

Contrary to chemical or other spectroscopic procedures the investigation of the spin exchange between stable spin probe radicals and the paramagnetic ^3^O_2_ by means of ESR has the advantage that the concentration can be determined by observing the changes in line widths [166], *i.e*., the x axis is precisely calibrated in units of frequency is used for finding the actual 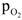. The marking of the mainly used nitroxyl spin probes with the isotope ^15^N instead of ^14^N is advantageous due to the increase in signal amplitude because the complete signal intensity is distributed over two lines only. Additional signal splittings can be avoided by substitution of ^1^H by ^2^H, which leads to smaller line widths. Altogether at comparable sensitivity the spin probe concentration can be reduced. Additionally the reduction of the dipole-dipole interactions between the probes leads to an increase of sensitivity of the 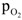. Disadvantageous seems to be the case that only one parameter (K [165]) is used which usually even depends on the applied radical concentration. Moreover, the signal-to-noise ratio decreases because the spectra are recorded with small modulation amplitudes. E.g., the reduced information content results already from the fact that out of 18 hyperfine lines only two are used.

In oximetry the influence of the spin-spin relaxation time T_2_ on the line widths of the spin probes with ^15^N and ^2^H marking the sensitivity could be increased.

In their considerations Halpern *et al*. [167] went further. They reduced the contribution of the inhomogeneous component of the spectrum and used the advantage of a selective deuteration for an additional reduction of the line width. In this they could find a compromise between the usage of the spectral “richness” and the reduction of the line widths. That compromise is of importance at low ESR frequencies (e.g., at 250 MHz) for in vivo investigations, including ESR imaging. E.g., after reduction of the contribution of the inhomogeneous component it could differentiated which part of line broadening results from interactions between the spin probe molecules themselves and the interactions with ^3^O_2_.

**Figure 15:**
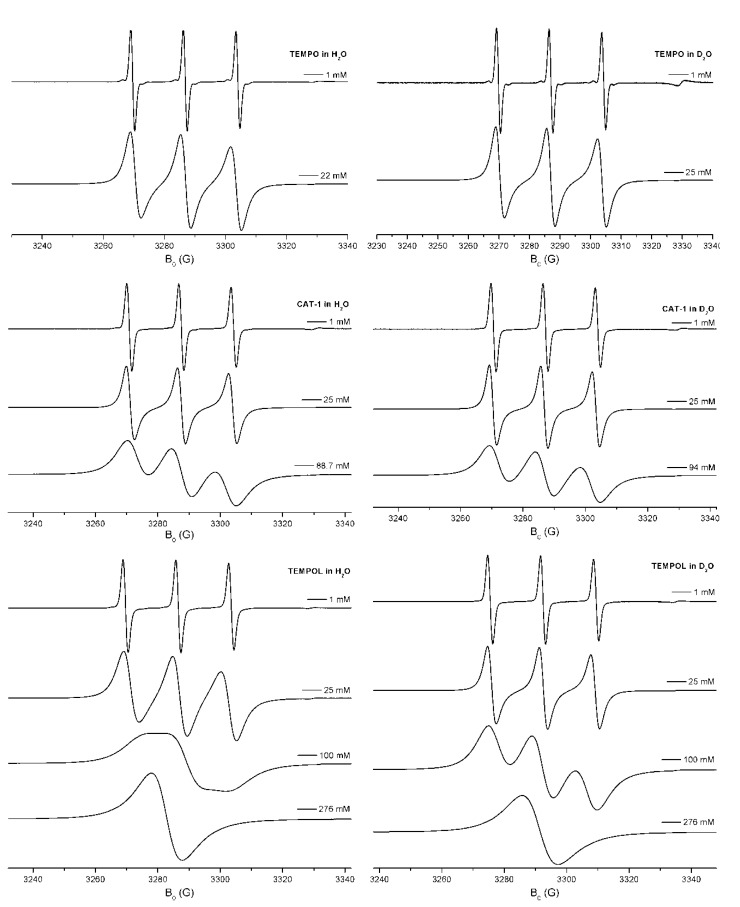
ESR spectra of different concentrations of the three spin probes TEMPO, CAT-1, and TEMPOL in H_2_O and D_2_O at 300 K.

The spin exchange of the dissolved spin probes TEMPO, CAT-1, and TEMPOL (see Figure 15) has been proven to be a valuable indicator for different interactions of the spin probes in H_2_O and D_2_O, respectively [96]. So, the different structure of the probes gave different rate constants k_e_ for the spin exchange at 293 K (contrary to the values of the hyperfine coupling which differ on slightly between H_2_O and D_2_O solutions) and make a differentiation between H_2_O and D_2_O possible. Different zero-point and dissociation energies for both fluid systems are the reason for that. Moreover, these disparities are responsible for other characteristic mainly macroscopic properties of water [168].

With respect to the influence of isotopes on chemical reactions further examples are discussed in [1,33,155,169,170,171]. Furthermore it is of interest how the spin exchange in biradicals can be elucidated by using the symmetrical and non-symmetrical isotope exchange [34].

### 3.9. Indication of Some Sources Dealing with Non-cw Techniques and Modern Applications of ESR

Non-stationary and multiple resonance techniques are described in [14] and [15]. Problems of the creation and handling of quantum bits are outlined in [172,173,174,175,176].

## 4. Conclusions

It is not only necessary to consider effects of naturally occurring isotopes for the interpretation of ESR spectra, but further information about spin density distribution, reaction behaviour and other phenomena of paramagnetic molecules, organic systems, and solids will be accessible.

Finally, the exchange of isotopes up to the synthesis of isotopically marked compounds is indispensable, despite the recent highly developed ESR technique. The conclusiveness of the interpretation is out of doubt. The variety of applications of isotopes extends via nearly all spin depending parameters, which provide a contribution to the ESR response. The relationship between electron spin and specific properties of isotopes such as nuclear spin, nuclear magnetic moment, mass and nuclear volume leads to characteristic phenomena such as electron spin, nuclear spin, vibronic couplings, tunnel effects and others which have a direct connection to the quantum mechanical basic effects. Well-planned experiments may approve the access to these phenomena.

Further developments of the ESR technique, the chemical methods for stabilizing reactive paramagnetic species, good command of isotopic marking on a chemical way, the adaption and improvement of quantum mechanical techniques for the interpretation of experimental findings will make it possible to follow the course of dynamic processes and chemical and biological reactions.
